# HIB/SPOP inhibits Ci/Gli-mediated tumorigenesis by modulating the RNA Polymerase II components stabilities

**DOI:** 10.1016/j.isci.2023.107334

**Published:** 2023-07-10

**Authors:** Yuxue Gao, Zhaoliang Shan, Chunhua Jian, Ying Wang, Xia Yao, Shengnan Li, Xiuxiu Ti, Guochun Zhao, Chen Liu, Qing Zhang

**Affiliations:** 1 State Key Laboratory of Pharmaceutical Biotechnology and MOE Key Laboratory of Model Animals for Disease Study, Jiangsu Key Laboratory of Molecular Medicine, Model Animal Research Center, School of Medicine, Nanjing University, Nanjing 210061, China; 2 Department of Medical Genetics, Nanjing Medical University, Nanjing 211166, China

**Keywords:** Genetics, Cell biology, Model organism

## Abstract

Hedgehog (Hh) signaling mediated by transcription factor Ci/Gli plays a vital role in embryonic development and adult tissue homeostasis in invertebrates and vertebrates, whose dysregulation leads to many human disorders, including cancer. However, till now, cofactors of Ci/Gli which can affect tumorigenesis are not well known. Here, through genetic screen, we find overexpression of active Ci alone is not sufficient to generate tumor-like eye phenotype in *Drosophila*, however, its overexpression combined with knockdown of *hib* causes a striking tumor-like big eye phenotype. Mechanistically, HIB/SPOP inhibits Ci/Gli-mediated tumorigenesis by modulating the RNA polymerase II (RNAPII) components Rpb3/Rpb7 stabilities in E3 ligase dependent manner. In addition, Ci/Gli can promote HIB/SPOP-mediated Rpb7/Rpb3 degradation. Taken together, our results indicate Ci/Gli needs to hook up with suitable RNAPII together to achieve the tumor-like eye phenotype and HIB/SPOP plays dual roles through controlling Ci/Gli and Rpb3/Rpb7 protein stabilities to temper Ci/Gli/RNAPII-mediated tumorigenesis.

## Highlights

• Overexpression of Ci alone is not sufficient to cause *Drosophila* eye tumorigenesis
• HIB inhibits Ci-mediated tumorigenesis in its E3 ligase dependent manner
• HIB degrades Rpb3/Rpb7 of RNAPII complex to inhibit Ci-mediated tumorigenesis
• SPOP and Gli2 play conserved roles to mediate hRpb7 degradation in mammalian cells

## Graphical abstract

**Figure:**
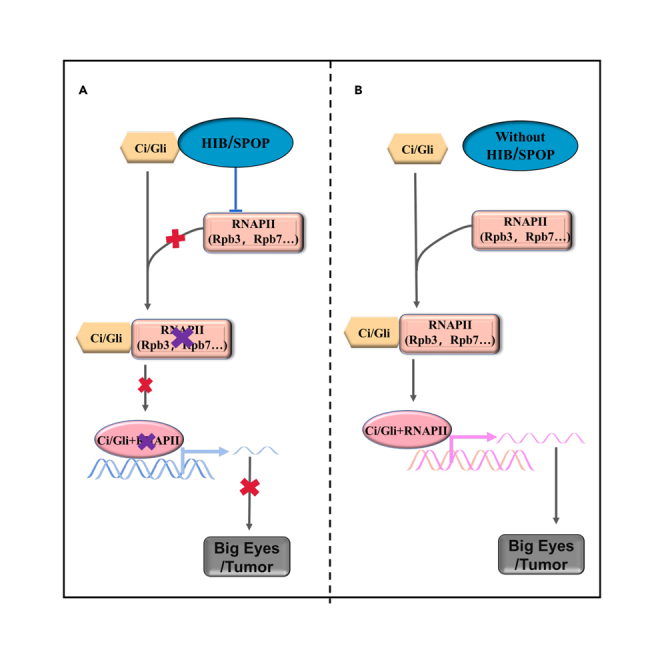

## Introduction

First identified in *Drosophila*, later shown to be largely conserved in mammals, the Hedgehog (Hh) family of secreted proteins plays a vital role in embryonic development and adult tissue homeostasis from invertebrates to vertebrates.^1^^,^^2^^,^^3^^,^^4^ Dysregulation of Hh signaling leads to many human disorders including birth defects such as holoprosencephaly, polydactyly, skeletal malformations, and kinds of human cancer, for example, basal cell carcinoma (BCC) and medulloblastoma (MB).^4^^,^^5^^,^^6^^,^^7^^,^^8^^,^^9^^,^^10^^,^^11^

In *Drosophila*, Hh pathway mainly consist of Hh ligand, twelve-span transmembrane protein Patched (Ptc), GPCR family of seven-span transmembrane protein Smoothened (Smo), kinesin family protein Costal-2 (Cos2), serine-threonine kinase Fused (Fu), the Suppressor of fused (Sufu), and zinc finger transcription factor Cubitus interruptus (Ci).^12^^,^^13^^,^^14^^,^^15^^,^^16^^,^^17^^,^^18^^,^^19^^,^^20^ In the absence of Hh, Ptc switches off the signaling by inhibiting Smo plasma membrane accumulation and activity.^21^^,^^22^^,^^23^^,^^24^^,^^25^^,^^26^^,^^27^^,^^28^^,^^29^^,^^30^ In this situation, Cos2 forms a complex with Fu, Ci, and Sufu, allowing its recruited protein kinase A (PKA), glycogen synthase kinase 3 (GSK3), and casein kinase 1 (CK1) to sequentially phosphorylate Ci. This hyperphosphorylated Ci further recruits F box protein Slimb of SCF (Skp1-Cullin1-F-box) ubiquitin ligase complex to proteolytically process the full-length Ci into a truncated form (CiR) that acts as a transcriptional repressor of Hh target gene *decapentaplegic* (*dpp*).^31^^,^^32^^,^^33^^,^^34^^,^^35^^,^^36^^,^^37^ In the presence of Hh, it dissociates Cos2/Ci complex, leading to blocking Ci phosphorylation, processing, and at the end turning Ci to be an active form (CiA) to promote Hh target gene *dpp*, *patched*(*ptc*) and *engrailed* (*en*) expression.^8^^,^^11^^,^^22^

As mentioned previously, Hh signaling-triggered human cancers are mediated by transcription factor Gli, including Gli1, 2, 3. Among them, Gli3 mainly functions as a repressor, while Gli1 and Gli2 as promoters.^4^^,^^11^^,^^38^ However, in some cases, only Gli is not sufficient to initiate tumorigenesis, the underlying mechanism and what cofactors of Ci/Gli affect tumorigenesis are not well known.

*hib* encodes Hh-induced BTB protein, which is also a Hh target gene in *Drosophila* wing discs. HIB together with Cul3 functions as an E3 ubiquitin ligase to modulate Ci degradation, forming a negative feedback loop to fine-tune Hh pathway activity.^39^^,^^40^ Speckle-type POZ protein (SPOP), which is a mammalian homologue of HIB, also modulates Gli2, Gli3 but not Gli1 protein levels.^41^^,^^42^ In humans, studies have shown that loss of function of SPOP mutation has closely related to many kinds of cancer, including glioma, prostate, breast cancer, etc.^43^^,^^44^^,^^45^^,^^46^^,^^47^ Why it can affect so wide spectrum tumorigenesis, the underlying mechanism is also not well known.

Here, we find overexpression of active Ci alone is not sufficient to generate tumor-like eye phenotype in *Drosophila*, however, its overexpression combined with *hib* RNAi causes a striking tumor-like big eye phenotype manifested by enlarged and protruded outward eye. Interestingly, in our Ci overexpression case, the occurrence of tumor-like eye is not due to *hib* RNAi-mediated upregulation of Ci level. Instead, it is related to the relative high RNA polymerase II (RNAPII) components Rpb3/Rpb7 levels. Our study demonstrates that except inhibiting Ci, HIB also synergistically inhibits RNAPII complex to prevent the occurrence of tumorigenesis. Further, we demonstrate SPOP is functionally conserved which can substitute HIB to inhibit Ci/Gli plus *hib* RNAi-mediated tumor-like eye phenotype.

## Results

### Genetic screen demonstrates that HIB inhibits ci-mediated tumor-like eye phenotype in *Drosophila*

**Figure 1:**
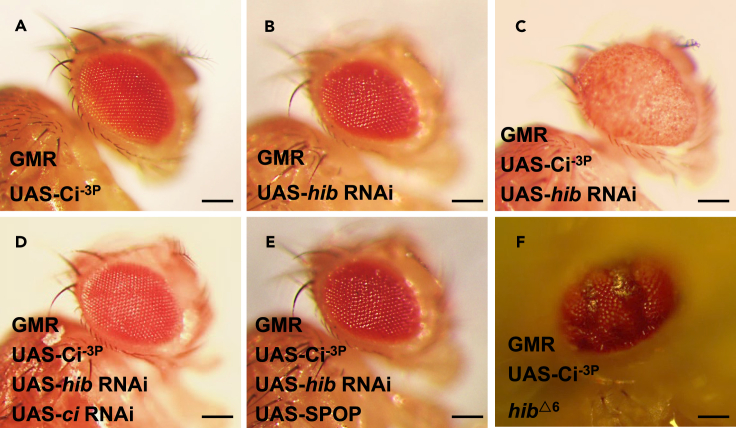
Co-overexpression of Ci^−3P^ and *hib* RNAi causes tumor-like eye phenotype in *Drosophila* (A–C) Co-overexpression of *hib* RNAi and Ci^−3P^, which is an active form of Ci with three PKA kinase sites mutated, led to tumor-like *Drosophila* eyes. (D and E) Knockdown of *ci* or co-expression SPOP could totally rescue the tumor-like eye phenotype. (F) Overexpressing Ci^−3P^ in *hib* clones also caused tumor-like eye phenotype. Scale bars: 2 mm.

Hh signaling-related tumorigenesis is mediated by transcription factor Ci/Gli, however, in some cases, only Ci/Gli is not sufficient to initiate tumorigenesis, the underlying mechanism and what cofactors of Ci/Gli affect tumorigenesis are not clear. To address this question, in *Drosophila,* overexpressing Ci^−3P^, which is an active form of Ci with three PKA kinase sites mutated,^48^ we found that Ci alone was insufficient to cause eye tumorigenesis (Figure 1A). Next, we overexpressed Ci^−3P^ and simultaneously knocked down around 7000 conserved genes between fly and human to screen Ci/Gli cofactors by checking the eye phenotype. In this first round of genetic screen, we got two hits, *hib*, and *warts*. Since knockdown of *warts* alone showed the similar phenotype with co-expression of Ci and *warts* RNAi (data not shown), we later gave up this hit. For *hib*, we found its knockdown alone showed normal eye phenotype, however, its knockdown together with overexpression of Ci^−3P^ caused very striking tumor-like eye phenotype (Figures 1A–1C). In this condition, either knockdown of *ci* or co-expression of mammalian homologue of HIB, SPOP, could completely inhibit the tumor-like eye phenotype (Figures 1D and 1E), indicating expression of Ci and inhibition of HIB are both necessary for making the tumor-like eye phenotype. Similarly, in *Drosophila* eye, we found that overexpressing Ci^−3P^ in *hib*^*−/−*^ mutant clones generated with FLP/FRT-mediated mitotic recombination also resulted in tumor-like phenotype inside *hib*^*−/−*^ clones (Figure 1F). Taken together, these results support the notion that Ci synergizing with *hib* RNAi causes the eye tumorigenesis. Of note, overexpression of HIB could not efficiently rescue the big eye phenotype owing it was eliminated by its powerful RNAi. To overcome this drawback, we overexpressed SPOP to substitute HIB to do the rescue experiment.

### Inhibition of Ci-mediated tumorigenesis is dependent on HIB/SPOP E3 ligase activity

**Figure 2:**
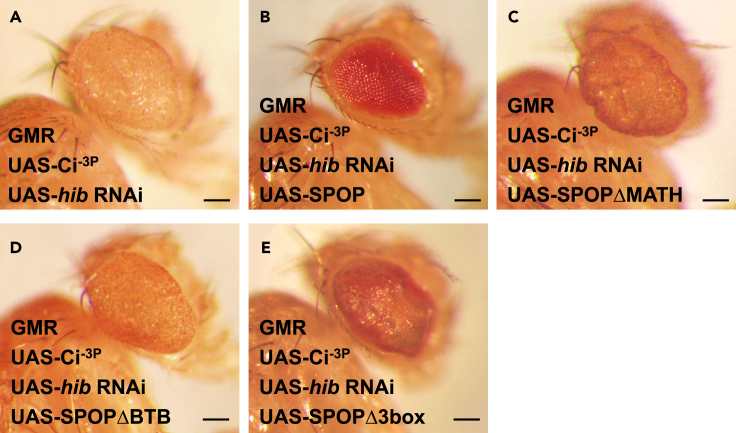
Inhibition of Ci-mediated tumor-like eye phenotype is dependent on HIB/SPOP E3 ligase activity (A–D) Lacking of MATH (C) or BTB (D) domain could not rescue the tumor-like eye phenotype caused by co-overexpression of Ci^−3P^ and *hib* RNAi with GMR Gal4. (E) Overexpression of SPOPΔ3box, which lacks of 299aa to 330aa could not rescue the tumor-like eye phenotype. Scale bars: 2 mm.

HIB/SPOP usually binds with Cul3 through its BTB domain (residues 190 aa–297 aa) to form an E3 ligase complex and recognizes substrates via its MATH domain (residues 28 aa–166 aa).^49^^,^^50^ To test whether HIB/SPOP E3 ligase activity is needed to inhibit the tumor-like eye phenotype, in co-overexpression of Ci^−3P^ and *hib* RNAi background, we first check the rescue effects of two SPOP E3 ligase-dead truncated forms, SPOPΔMATH and SPOPΔBTB, respectively. As shown in Figure 2, we found SPOP but not SPOPΔMATH and SPOPΔBTB could rescue the tumor-like eye phenotype, SPOPΔMATH and SPOPΔBTB even manifested the dominant negative effect shown by boosting the tumor-like eye phenotype (Figures 2C and 2D), indicating that both MATH and BTB domain are needed to inhibit the eye tumorigenesis. Next, we further test whether another SPOP E3 ligase mutant, SPOPΔ3box, can inhibit the tumor-like eye phenotype. SPOPΔ3box lacks of 299 aa to 330 aa, which decreases the association with Cul3 and weakens functional SPOP-Cul3 E3 ligase.^51^ We found that unlike SPOP, overexpression of SPOPΔ3box no longer rescued the tumor-like eye phenotype (Figure 2E). Taken together, these results suggest that HIB/SPOP inhibits the eye tumorigenesis dependent on its E3 ligase activity.

### The accumulation of ci alone is not sufficient to cause eye tumorigenesis

**Figure 3:**
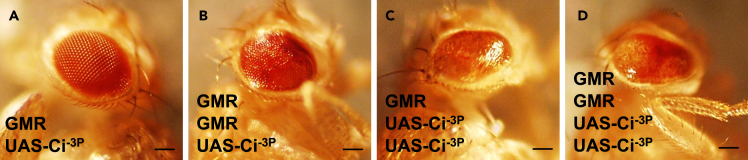
Ci^−3P^ alone is not sufficient to generate the tumor-like eye phenotype (A and B) One copy of Ci^−3P^ driven by one copy of GMR (A), or by two copies of GMR Gal4 (B) was not sufficient to generate the tumor-like eye phenotype. (C and D) Two copies of Ci^−3P^ driven by one copy of GMR (C), or by two copies of GMR Gal4 (D) were also not sufficient to generate the tumor-like eye phenotype. Scale bars: 2 mm.

Given HIB/SPOP modulates the tumor-like eye phenotype dependent on its E3 ligase activity, we next test whether the previous tumor-like eye phenotype caused by co-expression of Ci and *hib* RNAi is only due to *hib* RNAi-mediated upregulation of Ci level. Through modulating different copies of both Ci^−3P^ and GMR Gal4 to increase the amount of Ci^−3P^, we found that expression of one and two copies of Ci^−3P^ with one (Figures 3A and 3C) or two copies of GMR Gal4 (Figures 3B and 3D), none of them could cause tumor-like eye phenotype. Taken together, these results suggest that Ci^−3P^ alone is not sufficient to generate the big eye phenotype, which needs *hib* RNAi together to produce the tumor-like eye phenotype.

### Ci together with suitable Rpb3/Rpb7 of RNAPII complex mediates the tumorigenesis

**Figure 4:**
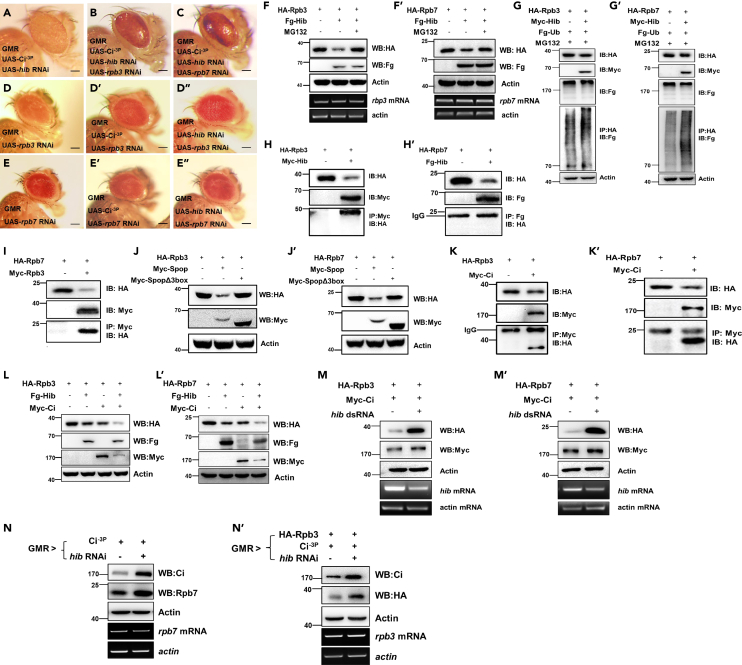
Ci and RNAPII complex together contribute to the tumor-like eye phenotype (A–E″) Knockdown of *rpb3* and *rpb7* could rescue the tumor-like eye phenotype. Scale bars: 2 mm. (F and F′) HIB degraded Rpb3/Rpb7 through proteasome. (G and G′) HIB could mediate Rpb3/Rpb7 ubiquitination. (H and H′) HIB bound Rpb3 but not Rpb7. (I) Rpb7 bound Rpb3. (J and J′) The degradation of Rpb3/Rpb7 by SPOP was dependent on its E3 ligase activity. (K–L′) Ci^−3P^ could bind Rpb3/Rpb7 and enhanced their degradation by HIB. (M–M′) In overexpression of Ci^−3P^ background, knockdown of *hib* dramatically upregulated Rpb3/7 levels in S2 cells. (N–N′) In overexpression of Ci^−3P^ background, knockdown of *hib* dramatically upregulated Rpb3/Rpb7 levels *in vivo.*

To unveil *hib* RNAi-affected factors which together with Ci mediate tumorigenesis, we did the second-round genetic screen in previous co-overexpression of Ci^−3P^ and *hib* RNAi tumor-like eye background. By crossing with the RNAi lines of around 7000 conserved genes between fly and human, we got several hits, including *rpb3*, *rpb7*, and *su(var)3–9*. Because HIB did not bind SuVar3-9 and affect its stability (data not shown), we ruled out the *su(var)3–9*. For *rpb3* and *rpb7*, we found knockdown of *rpb3* or *rpb7*, two subunits of the RNAPII complex, could quench the tumor-like eye phenotype (Figures 4A–4E″), suggesting that RNAPII machinery may be involved in mediating the tumor-like eye phenotype. Consistently, in S2 cells, we found HIB downregulated both Rpb3 and Rpb7 protein levels through proteasome (Figures 4F–4G′). Further IP experiments showed that HIB bound Rpb3 but not Rpb7, while Rpb3 bound Rpb7, suggesting that Rpb3 may recruit HIB to mediate itself and promote Rpb7 degradation (Figures 4H and 4I). Supporting this idea, we found co-transfection of Rpb3 indeed increased Rpb7 degradation (Figure 4I). Not surprisingly, SPOP also mediated Rpb3/Rpb7 degradation in its E3 ligase dependent manner (Figures 4J and 4J′). Given Ci binds HIB and also forms a transcription complex with RNAPII to mediate its target gene expression, we speculate that it may modulate HIB/Rpb3/Rpb7 complex, affecting HIB-mediated Rpb3/Rpb7 degradation. To test that, co-transfecting Rpb3/Rpb7 and HIB with or without Ci, we found that Ci bound Rpb3, Rpb7 and promoted their degradation by HIB (Figures 4K–4L′). Consistently, in S2 cells or *in vivo*, in overexpression of Ci background, knockdown of *hib* inhibited Rpb3/Rpb7 degradation (Figures 4M–4N′). Taken together, HIB may inhibit Ci-mediated tumorigenesis by downregulating Rpb3/Rpb7, suggesting both Ci and RNAPII complex together enable the occurrence of tumor-like eye phenotype.

### The tumor-like eye phenotype is related to excess interommatidial cells at pupal stage

**Figure 5:**
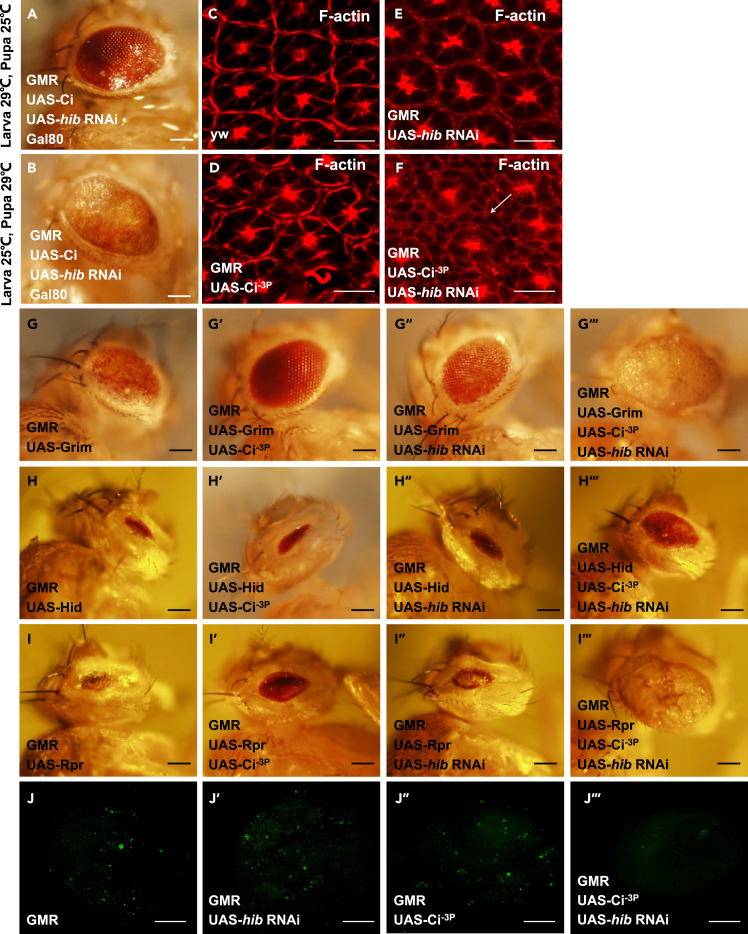
The tumor-like eye phenotype is related to excess interommatidial cells at pupal stage (A and B) Gal80 blocks Gal4 function at 25°C but not 29°C. Keeping 29 °C at the larva stage then switching temperature to 25°C in the pupal stage (A), or keeping 25 °C at the larva stage then switching temperature to 29°C in the pupal stage (B), only in case of (B), overexpression of Ci^−3P^ plus knockdown of *hib* with GMR Gal4 caused the tumor-like eye phenotype. Scale bars: 2 mm. (C–F) Compared with yw (C), Ci^−3P^ (D) or *hib* RNAi (E) alone, overexpression of Ci^−3P^and *hib* RNAi together showed excess interommatidial cells in pupal retinae stained with TRITC-labeled phalloidin that preferentially labels filamentous actin (F-actin) (F). Scale bars: 10 μm. (G–I‴) Ci^−3P^ synergizing with *hib* RNAi resisted the Grim/Rpr-mediated but not Hid-mediated apoptosis. Scale bars: 2 mm. (J–J‴) TUNEL staining (Green) was used to assess the status of apoptosis in a whole-mounted 25-h-APF retina. Compared with GMR (A), *hib* RNAi (B) or Ci^−3P^ (C) alone, co-overexpression of Ci^−3P^ and *hib* RNAi together (D) showed fewer apoptosis signals. Scale bars: 100 μm.

The tumor-like eye phenotype may be generated from over proliferation at the larval stage or dampened apoptosis during the pupal stage. To determine which stage is important for formation of tumor-like eye, we use Gal80 to block the GMR-driven co-overexpression of Ci^−3P^ and *hib* RNAi at different stages, the results showed that the tumor-like eye phenotype was generated at the pupal stage (Figures 5A and 5B). Our subsequent study revealed that compared with wild type (Figure 5C), Ci^−3P^ (Figure 5D) and *hib* RNAi (Figure 5E) alone, co-overexpression of Ci^−3P^ and *hib* RNAi (Figure 5F) presented excessive interommatidial cells, which are usually produced at the larval stage and then eliminated through apoptosis at the pupal stage for giving rise to the adult lattice. Collectively, the earlier results suggest that impaired apoptosis may be related to the tumor-like eye phenotype. Since Grim/Hid/Rpr-mediated apoptosis is involved in elimination of excessive interommatidial cells, next we check whether their mediated apoptosis was blocked upon co-overexpression of Ci^−3P^ and *hib* RNAi. As shown in Figures 5G–5I‴, co-overexpression of Ci^−3P^ and *hib* RNAi blocked Grim/Rpr but not Hid-mediated apoptosis, implying that the tumor-like eye may be related to disability of apoptosis mediated by Grim/Rpr but not Hid in our case. Of note, overexpression of Ci alone already blocked Grim-mediated apoptosis, but that did not produce any eye phenotype, indicating that Grim is also not responsible for the tumor-like eye phenotype mediated by the cooperation of both Ci and *hib* RNAi. For Rpr, we surprisingly found that overexpression of Ci or *hib* RNAi alone could not, but they together completely blocked Rpr-mediated apoptosis, suggesting Ci and *hib* RNAi synergistically achieve the tumor-like eye phenotype possibly partially through inhibiting Rpr-mediated apoptosis (Figures 5G–5I‴). Consistently, TUNEL assay showed compared with Ci^−3P^ and *hib* RNAi alone, co-expression of them inhibited apoptosis (Figures 5J–5J‴).

### SPOP and Gli2 can substitute HIB and ci to mediate the tumor-like eye phenotype in *Drosophila*

**Figure 6:**
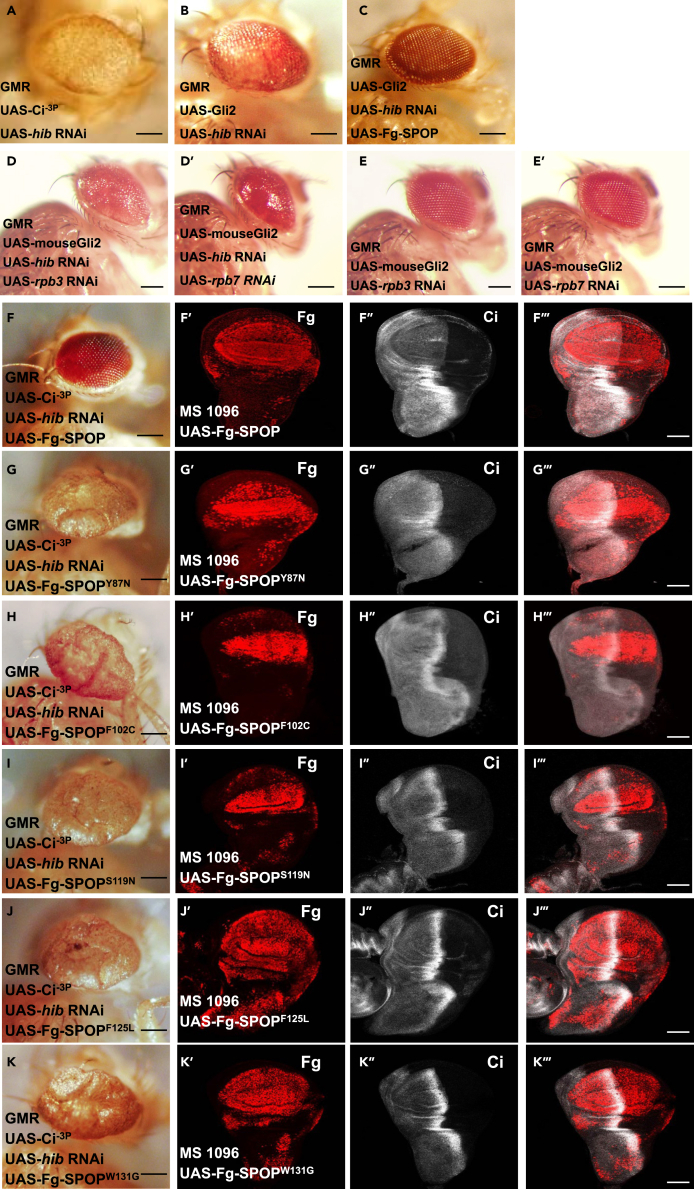
SPOP and Gli2 can substitute HIB and Ci to mediate the tumor-like eye phenotype in Drosophila (A) A big eye as the control. (B) Gli2 could substitute Ci to form tumor-like eye. (C) Overexpressing SPOP also could completely rescue Gli2-mediated the tumor-like eye phenotype. (D–E′) Knockdown *rpb3* or *rpb7* could rescue the Gli-mediated tumor-like eye phenotype. (A-E′ scale bars: 2 mm). (F–K‴) Among SPOP mutants, overexpression of SPOP^Y87N^ (G), SPOP^F102C^ (H), SPOP^S119N^ (I), SPOP^F125L^ (J) and SPOP^W131G^ (K), could not rescue the tumor-like eye phenotype. These SPOP mutants lacked of E3 ligase activities as shown by loss of the abilities to degrade Ci. (F′–F‴) Wing discs expressing Flag-SPOP with *MS1096* were immunestained with Ci^FL^ (red) antibody. The results showed that wild type SPOP degraded Ci. (F,G,H,I,J, and K scale bars: 2 mm). (G′–K‴) Overexpression of SPOP mutants such as SPOP^Y87N^ (G–G‴), SPOP^F102C^ (H–H‴), SPOP^S119N^ (I′–I‴), SPOP^F125L^ (J′–J‴) and SPOP^W131G^ (K′–K‴) could not degrade Ci. Scale bars: 20 μm.

**Figure 7:**
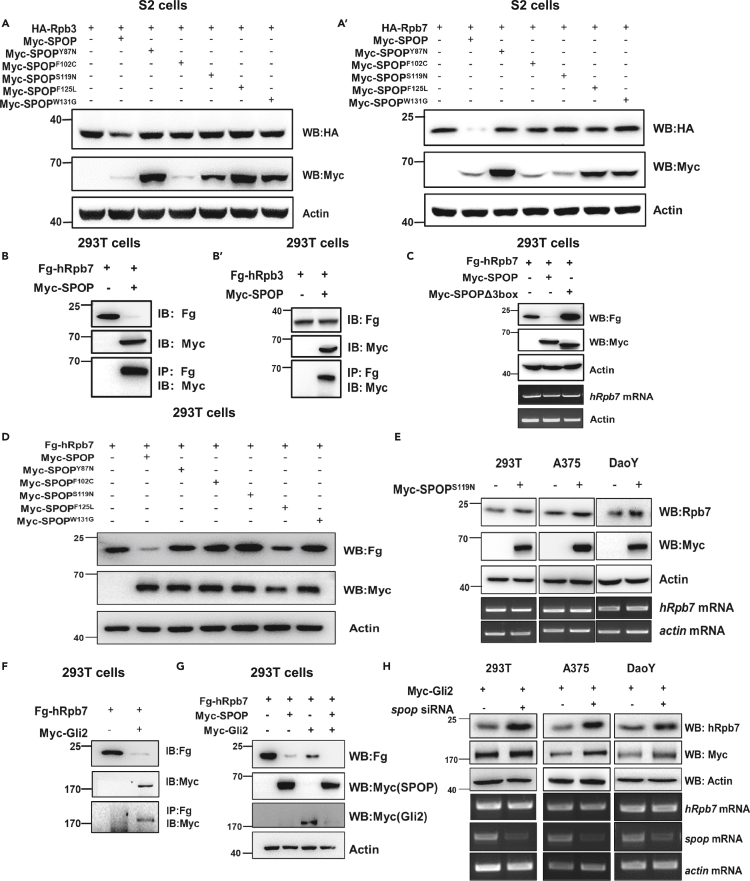
SPOP function is conserved in the regulation of RNAPII component hRpb7 stability in mammalian cells (A and A′) SPOP mutants which lack of E3 ligase activities could not degrade Rpb3/Rpb7 in S2 cells. (B and B′) SPOP could bind both hRpb3 and hRpb7 but only obviously degraded hRpb7 in mammalian 293T cells. (C) The degradation of hRpb7 by SPOP was dependent on its E3 ligase activity. (D) In 293T cells, SPOP mutants (SPOP^Y87N^, SPOP^F102C^, SPOP^S119N^, SPOP^F125L^, and SPOP^W131G^) did not degrade hRpb7. (E) In 293T cells and DaoY, A375 tumor cells, SPOP^S119N^ inhibited hRpb7 degradation. (F and G) Gli2 also bound hRpb7 and promoted hRpb7 degradation by SPOP. (H) In overexpression of Gli2 background, knockdown of *spop* upregulated hRpb7 levels in 293T cells and A375, DaoY tumor cells.

As mammalian homologue of HIB, SPOP is involved in various human cancers. In our previous study, we showed that SPOP function is evolutionally conserved in mediating Ci/Gli degradation. Consistently, using SPOP to replace HIB, it totally rescued the eye phenotype, confirming SPOP function is also conserved in mediating the tumor-like eye phenotype. In addition, we generated transgenic flies of several human cancer-related mutants of SPOP,^46^^,^^52^ including SPOP^Y87N^, SPOP^F102C^, SPOP^S119N^, SPOP^F125L^, SPOP^W131G^, which all prevented SPOP E3 ligase activity as shown by loss of the abilities to degrade both Ci (Figures 6G–6K‴) and Rpb3/Rpb7 (Figures 7A and 7A′). They no longer inhibited, instead even enhanced the tumor-like phenotype possibly due to their dominant negative effects (Figures 6G–6K), further indicating SPOP E3 ligase activity is necessary for the rescue of tumor-like eye phenotype. Similarly, Gli2 could substitute Ci together with knockdown of *hib* to cause the tumor-like eye phenotype (Figures 6A–6C). And knockdown *rpb3*/*rpb7* could also totally rescue the Gli2-mediated tumor-like eye phenotype (Figures 6D–6E′).

Next, in mammalian 293T cells, we found SPOP could bind both hRpb3 and hRpb7, but only obviously degraded hRpb7 in its E3 ligase dependent manner (Figures 7B and 7C). Contrastingly, cancer-related SPOP mutants no longer modulated hRpb7 stability (Figure 7D). Except 293T cells, in A375, DaoY tumor cells, taking SPOP^S119N^ as an example, it also inhibited hRpb7 degradation (Figure 7E). Like Ci, Gli2 also bound hRpb7 and promoted hRpb7 degradation by SPOP (Figures 7F and 7G). Consistently, in overexpression of Gli2 background, knockdown of *spop* dramatically upregulated hRpb7 levels in 293T cells and A375, DaoY tumor cells (Figure 7H). Taken together, all these results suggest that in *Drosophila,* Gli2 and SPOP can substitute Ci and HIB to mediate the same tumor-like eye phenotype, in mammalian cells, hRpb7 stability is similarly regulated by Gli2 and SPOP, indicating that our model and the identified rescue genes are useful for future study of Hh- and SPOP-related tumorigenesis.

## Discussion

Hh signaling transduced by transcription factor Ci/Gli is essential for embryonic development and adult tissue homeostasis across species. Its dysregulation links with many kinds of human disorders, including various types of cancer, however, till now, cofactors of Ci/Gli which affect tumorigenesis are not well known. In this study, we find overexpression of active Ci alone is not sufficient to generate a tumor-like eye phenotype in *Drosophila*, however, its overexpression combined with knockdown of *hib* causes the striking tumor-like eye phenotype. Subsequent results indicate HIB regulates this phenotype dependent on its E3 ligase activity. Considering HIB can function as an E3 ligase of Ci, we first test whether *hib* RNAi mediates the phenotype only by boosting Ci accumulation. The results indicate it is not the case, since only increasing Ci is not sufficient to initiate the tumorigenesis. Taking advantage of our tumor-like eye fly model, we finally find HIB inhibits Rpb3/Rpb7 to prevent the tumor-like eye phenotype. Rpb3 and Rpb7, as two subunits of RNAPII complex, function in the same manner, suggesting HIB regulates the phenotype through modulating RNAPII machinery and the levels of RNAPII subunits are important for together with Ci to generate the tumorigenesis.

Why overexpression of Ci alone is not sufficient to mediate tumorigenesis but it plus knockdown of *hib* can? No matter HIB is expressed as Ci target gene (in *Drosophila* wing disc) or not (in the posterior region of *Drosophila* eye disc), in the presence of HIB, on the one hand, it can downregulate Rpb3/Rpb7 to inhibit RNAPII function independent on Ci. On the other hand, Ci promotes HIB-mediated Rpb3/Rpb7 degradation, resulting in RNAPII machinery working inefficiently. Therefore, only Ci overexpression case, overall high Ci with relatively low RNAPII machinery does not work effectively to generate tumor-like phenotype. However, co-overexpression of Ci and *hib* RNAi not only further increases Ci level but also lifts the burden on RNAPII machinery by stabilizing its subunits, therefore, leading to achieving the tumor-like eye phenotype more easily and effectively.

As mentioned, SPOP is involved in kinds of human cancer. The reason why it is related to a wide spectrum of tumors is not clear. Based on our study, dysfunction of SPOP will upregulate transcription machine RNAPII levels, leading to relatively easily reaching the thresholds of specific tumorigenesis. Additionally, except Ci/Gli, defective SPOP may also upregulate the levels of some other specific transcription factors which are usually degraded by SPOP. Taken these two aspects together, loss of function of SPOP may upregulate both specific transcription factors and general RNAPII machinery levels, leading to reaching the thresholds of various SPOP-regulated transcription factors-driven cancers easily.

As mammalian homologue of Ci, Gli includes Gli1, Gli2, and Gli3. Among them, Gli1 itself is also an Hh target gene, which and Gli2 mainly function as activators, while Gli3 as a repressor. It is reported that in some cases, loss of function of *Ptch1* (*Ptch1*^−/−^) and gain of function of Smo (*SmoM2*) effectively induce MB, but overexpression of active Gli2 alone is not sufficient, the underlying reason is not clear.^53^ Based on our study, any essential subunit dysfunction will impair the function of the whole RNAPII complex. We think in the context with SPOP, SPOP degrades the subunit hRpb7 of RNAPII, leading to forming low level functional RNAPII complex. Additionally, overexpressed Gli2 further promotes degradation of hRpb7, leading to forming low level functional Gli2/RNAPII complex according to limited Rpb7 level but not the overexpressed Gli2 level, subsequently causing correspondent low level Gli1/RNAPII complex. Therefore, high level overexpressed Gli2 with non-matched low level RNAPII generates low level functional Gli2/RNAPII and Gli1/RNAPII complexes, which is not easy to reach the needed threshold to effectively trigger MB. However, even *Ptch1*^−/−^ and *SmoM2* activate Gli2, whose level is lower than overexpressed Gli2, therefore, it mediates relatively weak inhibition on RNAPII. Consequently, this leads to a little higher RNAPII, therefore, resulting in forming correspondent a little higher functional Gli2/RNAPII and Gli1/RNAPII complexes. But such little increase may be still not enough to effectively trigger MB. Except this, *Ptch1*^−/−^ and *SmoM2* also inhibit Gli3’s repressor function, this may decrease MB threshold or further tip the balance to trigger MB. Taken together, these results imply that Gli1/2/3, all streams may function together to effectively fulfill induction of MB. In contrast to previous case, in the context without SPOP or SPOP dysfunction, RNAPII maintains relative high level, leading to forming relative high level Gli2/RNAPII complex, which consequently causes correspondent high level Gli1/RNAPII. Therefore, they together may already reach the threshold of tumorigenesis. In this case, we predict *Ptch1*^−/−^, *SmoM2* and overexpression of active Gli2 alone all may effectively cause tumorigenesis, including MB.

As for why overexpression of Ci together with knockdown of *hib* antagonizes the apoptosis mediated by Rpr, but either overexpression of Ci or knockdown of *hib* alone not, the underlying mechanism is elusive. The specificity is possibly due to that overexpression of Ci plus knockdown of *hib* forms high level Ci/RNAPII complex which surpasses the specific transcription threshold needed for antagonizing Rpr-mediated apoptosis or high Ci/RNAPII may selectively express Ci target genes needed to fulfill the distinct roles compared with their alone. Of note, simultaneous overexpression of Ci and DIAP1 or P35 which blocks apoptosis did not cause tumor-like eye phenotype (Figure S1), suggesting Ci and *hib* RNAi together-mediated tumor-like eye phenotype may be only partially through inhibiting apoptosis, except that, other unidentified process must be involved in driving the big eye phenotype.

Finally, SPOP and Gli2 can substitute HIB and Ci to mediate the same tumor-like eye phenotype in *Drosophila*, in mammalian cells, hRpb7 stability is similarly regulated by Gli2 and SPOP, indicating that they are functionally conserved. Our study demonstrates SPOP is a very important cofactor of Ci/Gli, which plays a dual role in regulating both Ci/Gli and RNAPII machinery for suitable Hh signaling outcome, its loss of function will unleash Ci/Gli and RNAP II machinery to cause abnormal high Hh signaling or parallel signaling, leading to various Hh-related tumorigeneses. Accordingly, this mechanism is also applied to SPOP-regulated other transcription factors-related kinds of cancer.

### Limitations of study

As mentioned previously, HIB/SPOP inhibits Ci/Gli-mediated tumorigenesis by modulating the RNAPII components Rpb3/Rpb7 stabilities and Ci/Gli can promote HIB/SPOP-mediated Rpb7/Rpb3 degradation. However, the detail underlying mechanism needs to be further addressed, for examples, except apoptosis inhibition, what is other unidentified process involved in driving the big eye phenotype? Except Rpb3/Rpb7, whether other RNAPII complex components are regulated by HIB/SPOP needs to be checked. In addition, we indicate SPOP is a very important cofactor of Ci/Gli, which plays a dual role in regulating both Ci/Gli and RNAPII machinery, how HIB/SPOP-RNAPII axis affects Ci/Gli-mediated tumorigeneses needs to be further investigated.

## STAR★Methods

### Key resources table

**Table:** 

REAGENT or RESOURCE	SOURCE	IDENTIFIER
**Antibodies**		

Rabbit monoclonal anti-hRpb7	Origene	Cat#TA890034
Rat monoclonal anti-Ci	DSHB	Cat#2A1; RRID: AB_2109711
Mouse monoclonal anti-HA	Santa Cruz	Cat# sc-7392; RRID: AB_627809
Mouse monoclonal anti-Myc	Santa Cruz	Cat#sc-40; RRID: AB_2892598
Mouse monoclonal anti-Flag	Sigma	Cat#F4049; RRID: AB_439701
Mouse monoclonal anti-Actin	Genscript	Cat#A00702; RRID: AB_914102
Goat anti-mouse Ig-G antibody	Jackson ImmunoResearch	Cat#115-545-003; RRID: AB_2338840

**Critical commercial assays**		

TRITC-labeled phalloidin	Sigma	Cat#FAK100
TUNEL Apoptosis Assay Kit	Beyotime	Cat#c1086
lipofectamine 2000	Invitrogen	Cat#11668019
Chemiluminescent detection kit	GE healthcare	Cat#RPN2134

### Resource availability

#### Lead contact

Further information and requests for resources should be directed to and will be fulfilled by the lead contact, Qing Zhang (zhangqing@nju.edu.cn).

#### Materials availability

This study did not generate new unique reagents.

#### Data and code availability

• The data reported in this paper will be shared upon request to the lead corresponding author (zhangqing@nju.edu.cn).
• This paper does not report original code.
• Any additional information required to reanalyze the data reported in this paper is available from the lead contact upon request.

### Experimental model and study participant details

#### Cell lines

The *Drosophila* embryonic cell line S2 were obtained from Yun Zhao lab (CAS Center for Excellence in Molecular Cell Science, Shanghai, China) and was cultured in the Schneider’s *Drosophila* Medium (Invitrogen) with 10% fetal bovine serum (FBS, Gibco),100 U/ml of penicillin and 100 μg/mL of Streptomycin (P/S, Gibco) in a humidified incubator at 28°C. The human embryonic kidney cell line 293T and human melanoma cell line A375 were obtained from Ying Cao lab (Nanjing university, Nanjing, China). The human medulloblastoma cell line DaoY was obtained from Chen Liu lab (Nanjing Medical University, Nanjing, China). They were cultured in Dulbecco’s modified Eagle’s medium (DMEM, HyClone), with 10% fetal bovine serum (FBS, Gibco),100 U/ml of penicillin and 100 μg/ml of Streptomycin (P/S, Gibco) in a humidified incubator with 5% CO_2_ at 37°C.

#### Animal model

The RNAi lines that targeted *ci* (2125R-1), *hib* (9924R-1), *rpb3* (7885R-1) and *rpb7* (v100309) used in genetic screen were obtained from the National Institute of Genetics Stock Center (NIG), Japan and the Vienna Drosophila RNAi Center (VDRC), Austria. eyflp, hsflp and FRT82 flies were obtained from the Bloomington Stock Center. Flies of MS1096, GMR-Gal4, Gal80, HA-Ci^−3P^, Fg/Myc-SPOP, Fg-SPOPΔ3box, Fg-SPOPΔMATH, Fg-SPOPΔBTB, Myc-mouse-Gli2 have been described.^40^^,^^54^ *hib*^*Δ6*^ is a *hib* mutant allele, whose coding sequence is replaced by the white gene. The transgenic flies of Fg-Rpb3, Fg-Rpb7, HA-Grim, HA-Hid, HA-Rpr, Fg-SPOP^Y87N^, Fg-SPOP^F102C^, Fg-SPOP^S119N^, Fg-SPOP^F125L^, Fg-SPOP^K129E^, Fg-SPOP^W131G^ and Fg-SPOP^F133V^ were generated by injection of corresponding constructs into Drosophila embryos according to the methods described previously.^55^ The parental strain for all germline transformations is w1118. All stocks used in this study were maintained and raised under standard conditions.

### Method details

#### Genetic screen

The RNAi lines were obtained from the National Institute of Genetics Stock Center (NIG), Japan and the Vienna *Drosophila* RNAi Center (VDRC), Austria. We totally screened around 7000 RNAi lines which target to the conserved genes between *Drosophila* and Mammals. We did the first and the second round of genetic screening in GMR Ci^−3P^ and in GMR Ci^−3P^/*hib* RNAi background, respectively.

#### Plasmid constructs

The constructs for S2 cell transfection experiments are as follows: HA/Myc-Rpb3, HA/Myc-Rpb7, Myc/Fg-HIB, HA-Grim, HA-Hid, HA-Rpr and Myc/Fg-SPOP, their corresponding cDNA fragments were amplified and cloned into the pUAST vectors. Similarly, the constructs of Fg-hRpb3, Fg-hRpb7, Myc-hGli2, Myc-SPOP and Myc-SPOPΔ3box used in mammalian cells were cloned into the pcDNA3.1 vectors. The mutants of Myc/Fg-SPOP, including Fg/Myc-SPOP^Y87N^, Fg/Myc-SPOP^F102C^, Fg/Myc-SPOP^S119N^, Fg/Myc-SPOP^F125L^, Fg/Myc-SPOP^K129E^, Fg/Myc-SPOP^W131G^ and Fg/Myc-SPOP^F133V^, were made through the PCR-based site-directed mutagenesis and cloned into the pUAST and pcDNA3.1 vectors, respectively.

#### Immunostaining

Immunostaining of imaginal discs were performed with standard protocols.^56^^,^^57^ To immunostain the pupal *Drosophila* visual system, samples should be dissected after 48h when third-instar larvae begin to enter pupation. Then the whole visual system is held in fixative and primary antibody solution with shaking in overnight at 4°C. After adding the secondary antibody, the Drosophila pupal visual system can be mounted for confocal microscopy (TCS SP5II, Leica Company, Germany). Antibodies were used in this study: rat anti-Ci (2A) (1:50; DSHB); mouse anti-Flag (M2) (1:200; Sigma); mouse anti-HA (F7) (1:200; Santa Cruz); TRITC-labeled phalloidin (1:250; Sigma).

#### TUNEL assay

Pupal retinas were dissected in PBS and fixed for 30 min in 4% paraformaldehyde. The apoptotic cells were determined by TUNEL staining using a One Step TUNEL Apoptosis Assay Kit (Beyotime, Jiangsu, China). Tissues were incubated at 37°C for 1 h in the mixture of enzyme and fluorescent labeling solution then rinsed in PBS. Images were captured using a confocal microscopy (TCS SP5II, Leica Company, Germany).

#### Cell-based ubiquitination assay

For cell-based ubiquitination assays, cells were lysed with denaturing buffer (1% SDS, 50 mM Tris-base, pH 7.5, 0.5 mM EDTA, and 1 mM DTT) and incubated at 100°C for 5 min. The lysates were then diluted 10-fold with regular lysis buffer (50 mM Tris pH8.0, 0.1 M NaCl, 10 mM NaF, 1 mM Na3VO4, 0.5% NP-40, 10% Glycerol and 1 mM EDTA pH8.0) and subject to IP and IB analysis.

#### Generating clones of mutant cells

Clones of mutant cells were generated by FLP/FRT-mediated mitotic recombination as described.^31^^,^^40^ Genotypes for generating clones are as follows: *hib* clones in eye with expressing UAS transgenes: *ey-flp*; (*GMR-Gal4*/*UAS-Ci*^*−3P*^); FRT82 *hib*/FRT82 *Gal80*.

#### Cell transfection, Western blot assays

Transfection of S2 cells were carried out using calcium phosphate transfection method. Usually, S2 cells are transfected in 10 cm plates with no more than 20 μg of total DNA for a *ubiquitin-Gal4* construct and other co-transfected pUAST expression vectors. 36–48 h after transfection, cells are harvested for Western blot analysis with standard protocols as previously described. 293T cells were transfected using lipofectamine 2000 (Invitrogen) according to the manufacturer’s instructions. 48 h after transfection, cells were harvested for immunoprecipitation and Western blot analysis with standard protocols as described.

The primary antibodies used were mouse anti-HA (F7) (1:5000; Santa Cruz); mouse anti-Myc (9E10) (1:2500; Santa Cruz); mouse anti-Flag (1:2500; Sigma) and mouse anti-Actin (A00702) (1:5000: Genscript). After incubation with HRP-coupled secondary antibodies (goat anti-mouse diluted 1:10000, Jackson ImmunoResearch), the blots were visualized using a chemiluminescent detection kit (GE healthcare).

### Quantification and statistical analysis

The data shown in all figures were representative of three or more independent experiments.
