# Supplementary material for: HIB/SPOP inhibits Ci/Gli-mediated tumorigenesis by modulating the RNA Polymerase II components stabilities
Source: iScience. 2023 Jul 10;26(8):107334. doi: 10.1016/j.isci.2023.107334 (PMC10404538; doi:10.1016/j.isci.2023.107334)
Supplement: Document S1. Figure S1 [file mmc1.pdf]

iScience, Volume *26*

Supplemental information

HIB/SPOP inhibits Ci/Gli-mediated tumorigenesis by modulating the RNA Polymerase II components stabilities

Yuxue Gao, Zhaoliang Shan, Chunhua Jian, Ying Wang, Xia Yao, Shengnan Li, Xiuxiu Ti, Guochun Zhao, Chen Liu, and Qing Zhang

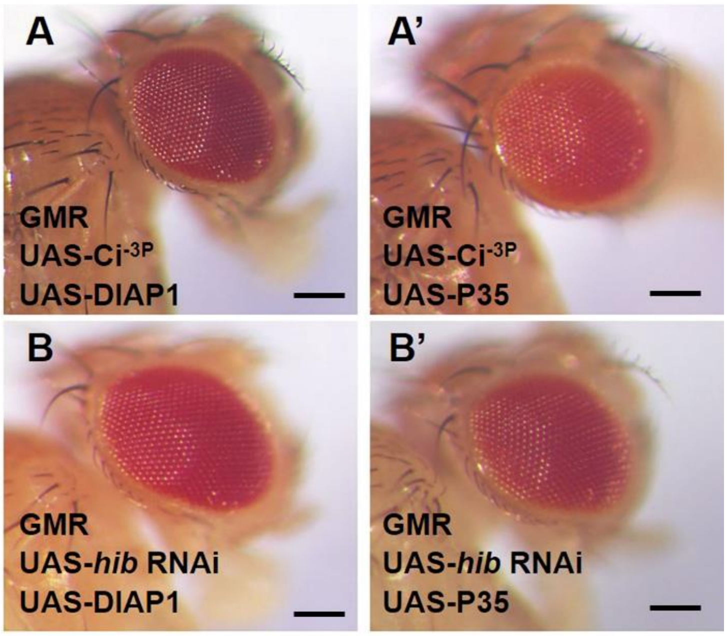

Figure S1. Simultaneous overexpression of Ci or *hib* RNAi with DIAP1 and P35 does not cause the tumor-like eye phenotype, Related to Figure 5

(A and A') Co-expression Ci^{-3P} with DIAP1 and P35 could not cause tumor-like eye phenotype.

(B and B') Overexpression *hib* RNAi with DIAP1 and P35 could not cause tumor-like eye phenotype.

Scale bars, 2 mm.
